# Under renovation: Large-scale societal events induce shifts between moral ideologies

**DOI:** 10.1371/journal.pone.0336520

**Published:** 2025-12-10

**Authors:** Yen-Ping Chang, Yu-Shan Chiang, Chun-Kun Wang

**Affiliations:** 1 Department of Educational Psychology and Counselling, National Tsing Hua University, Taiwan; 2 School of Psychological Sciences, University of Tasmania, Australia; 3 School of Education, Indiana University Bloomington, United States of America; 4 Oracle America, INC., Texas, United States of America; University of Malta, MALTA

## Abstract

Existing research has revealed various cross-sectional inter-personal/group (e.g., regional) differences in moral traits; it has, however, paid less attention to cross-temporal intra-personal/group moral differences – simply, changes over time. Addressing the intellectual gap, the present research proposes that morality is redefined – renovated – in times when a society undergoes widespread social upheavals, in a manner that the incoming new ideology would better serve the society. Using longitudinal social media (Reddit) text data (N = 459,077,063) from Reddit over the 63 months since the last global great recession starting in late 2007, we report a partial, economy-focused demonstration of the hypothesis, wherein people associated their overall conception of morality more with support for authority and hierarchy and less with fairness and equality when the economic stress was increasing. In comparison, the public reversed the association to lean back on fairness and away from authority when the market was on the rise. Together, the findings offer insights into the communal nature of morality and call for caution that personal moral disposition may be malleable and fluctuating with the collective surroundings, thus cannot be adequately measured without first considering what the disposition means for the cultural-social unity at the moment.

## Introduction

Should a country balance wealth and redistribute resources from the haves to the have-nots? Or should it protect the right to property, thus for those who actually possess something to protect? What is fairness between both ends of seemingly equally justifiable societal needs? Traditionally, scientists tackle moral, right-versus-wrong judgment about social matters by measuring individuals’ moral dispositions manifested in actions or attitudes, e.g., support for resource redistribution [e.g., 1,2]. This approach is commonly carried out cross-sectionally with self-report personality assessments [e.g., 3,4], which may imply that moral decision-making in everyday life can naturally be, though surely not merely, a personality trait rather stable and not varying much over time [5].

Notwithstanding the above interpretation of morality, take the attitude toward sexual intercourse. Seemingly mundane and well-understood, the behavior has been passionately yet contradictorily argued to be *immorally* corrupting (i.e., should be normatively suppressed; see [6], *amorally* elective [i.e., should be let along from a normative control), and *morally* energizing [i.e., should be normatively encouraged; see 7]. And it is easy to see how the contradiction arose: The competing stances resulted not (only) from the disagreeing parties speaking from disconnected geo-socio-locations at a given time, but (also) from distinct temporal positions *in* time. The proponents and their ideas lived in separate historical periods and ethe. Moral judgment here and likely everywhere may be an issue of longitudinal shifts as much as static propensities.

Of course, we have no intent to suggest that the present research will be the first to examine moral changes quantitatively. Some emerging work has started doing that [e.g., 8], let alone the long qualitative literature on the topic [e.g., 9]. What we would like to highlight, instead, is that the existence of moral changes could be both a substantive and a methodological issue, thus resulting in two kinds of moral shifts being confused in the literature. Specifically, in the existing investigation into social-cultural shifts in moral disposition, the popular methodology is to track the long-term fluctuations in people’s (dis)approval of a focal assumed-to-be-moral disposition X *per se* [e.g., 8,10], describing the approval’s ups and downs over time. However, if the extent to which X is deemed moral is itself shifting – the second kind of changes, which will be called renovations below to avoid confusion – it then becomes necessary to explicitly assess and continuously monitor such a degree of the “moralization of X” (perceiving X as morally relevant) or, conversely, the “demoralization” of X (decoupling X from morality and making it morally irrelevant). The moral relevance of X cannot be simplistically assumed as a constant. Measuring the alignment with X per se, in turn, would not capture the true shifts in moral disposition in this situation. Simply, when only such alignment (e.g., with temperance) is measured, people could appear to turn immoral for demonstrating a decrease in such measure on attitude or behavior (e.g., drinking). In reality, they might nevertheless experience little or even the opposite moral development in the eyes of others as well as themselves, due to a termination or reversion of the association between X and the overall idea of morality [relatively, drinking is much less deemed evil than unhealthy today; 11].

To make the case, our paper proceeds as follows. We introduce the functionalist approach to morality, which postulates that morality is first and foremost formed for the collective as a unity to survive its socio-ecological environment. Given that the environment reshapes itself and poses distinct challenges at different times, it is subsequently inferred that morality would morph, with some but not all of its components emphasized and morally enforced, at one point but not others, so the updated morality can fit with the changing world. To demonstrate this continuous moral *renovation*, we also follow up with an empirical analysis of the public’s fluctuating morality over the 2009 global Great Recession.

### Making moral judgment

Though theorists still debate the exact dimensions underlying morality, they generally agree that it must comprise a limited number of fundamental dimensions [12–14]. In this multi-dimensional space, people occupy different locations, holding various combinations of the levels of the dimensions. Those distinct combinations thus give rise to the individuals’ moral ideologies, much like how dissimilar dishes (cf full-blown moral idiologies) are ways of mixing a few similar essential ingredients (cf foundational moral dimensions).

Crucially, though much past research studied morality in terms of whether individuals find a target moral or *immoral*, the understanding of morality as multi-dimensional would suggest that moral judgment at the moral-foundations level is likely an issue of comparing the moral versus the *amoral* [15,16]. Take same-sex marriage, superficially moral for some and *immoral* for others. Existing work suggests that people oppose same-sex bonding because they weigh the purity of the traditional familial structure highly, sidelining “without denying” the care for same-sex persons [16]. Care is not immoral, but merely silenced and made *amoral*. It is indeed unimaginable that anyone would find kindness in and of itself wrong. Likewise, it is documented that others advocate for same-sex marriage out of a higher moral weight assigned to care than to purity, thus overlooking but not objecting to the latter [16]. Both the pro- and the anti-same-sex-union ideologies endorse care and purity as not immoral. The two sides arrive at opposite conclusions when they each determine that only one of the two foundations is not amoral.

It is subsequently logical that not only may people take different positions in the moral space at a given moment, showing cross-sectional *between-individual* differences; they may move in the space and differ from themselves from time to time, showing cross-temporal *within-individual* changes. Here, we test this latter possibility of longitudinal personality shifts with the five-dimensional version of the Moral Foundations Theory [MFT; 13].

### MFT and Contextual changes cause moral ideology renovations

The MFT suggests morality consists of five basic dimensions, or, foundations. They are *care* (v. harm) for others, *fairness* (v. cheating) in interactions, *loyalty* for (v. betrayal of) in-groups, supporting (v. subverting) *authority* within the groups, and *purity* (v. degradation) of oneself [17,18]. While searching for the basic ingredients of morality as do most other popular frameworks [e.g., 14,19], in addition to depicting what morality is/has, the MFT is set apart for postulating *social functionalism* for how morality forms. That is, a moral foundation takes shape and becomes morally relevant and socially normalized when the public has a shared need for the norm to survive socio-ecological stressors together as a collective [18,20]. This group-focused approach, in turn, allows us a vantage point to more easily – if not exclusively – peek into the larger-scale, societal dynamics of morality than would more individual-focused approaches. In particular, though the literature often investigates the construction of morality along cross-sectionally (e.g., geographically) varying environmental challenges across places [21], it can be derived from the MFT that morality may also be a social *re*-construction that tracks with environmental *shifts* within one place. There, to realize its supposed survival utility according to social functionalism, the fitting morality will need to be acknowledged by a sufficient number of individuals for it to be socially normed.

Together, with individual differences, group members should bear commonality and move somewhat alongside during the hypothesized moral renovation. Particularly, we reason that large-scale ecological and societal happenings, such as natural or societal havoc, would most easily induce such collective moral reconstruction. It is because, at its extension, the upheaval shall equally and unequivocally inform the people about their common fate [22]. The challenge will, therefore, be addressed, with the corresponding moral foundations taken out of the toolbox, emphasized, and normalized across the public.

Technically, our reasoning translates to a serial association from large-scale events, to perceived changes in environmental stressors, and then perceived needs to redefine morality as a more functional mixture of its foundations. Because the last piece in this chain describes associations – between the constituting foundations of morality and the overall idea of what morality is – analytically, the series suggests a model of moderation [cf mediation) whereby people’s general conception of morality fluctuates to depend on foundation X relative to other foundations, manifesting X’s momentary moral relevance, when an eco-societal change signals to the group it would benefit from the function of X.

### The current research

For an empirical demonstration of the proposed moral reconstruction, we derive that, facing resource scarcity, e.g., in economic depression, the public would particularly moralize – associate general morality with – the foundation of authority. Alternatively, when the collective is out of depression and the economy is reviving, the antagonist foundation – fairness – would be moralized. Our reasoning follows the functionalist assumptions that authority legitimizes disparity and justifies punishing dissidents when the stratification is steep and the social hierarchy is about to tear [23,24]. Conversely, fairness stops the haves on the higher rungs of the societal ladder from exploiting the have-nots on the lower ones [17,18]. The two foundations are directly economic, operating against, therefore dynamically balancing each other in sustaining the collective.

Here, it might be worth noting that, different from most existing work, we are not suggesting societal changes would induce the endorsement of moral foundations – the main effects. People may pursue fairness for selfish, thus amoral reasons; studying fairness *per se* is not enough. Instead, our study focuses on the explicit moralization of the foundations, reflected in their contributions to general morality, and how the pattern covaries with temporary events – the time-moderated effects.

Moreover, we narrowed in on the economy for a practical reason: the data. Besides the economy’s profound impacts, it has the most meticulously collected and representative records. Pulling together such records with the big, naturalistic data on social media containing everyday moral discourse (see Method for details), the present study would consequently offer a statistically powerful – albeit economy-specific – demonstration for our overall theorizing.

## Method

We assessed whether the natural field experiment created by the 2009 global Great Recession elicited the proposed moral renovation in an online community (post N = 459,077,063), over the years when the economic disaster started in late 2007, peaked in 2009, and then gradually subsided until late 2012. Given the nature of the recession, we focused on the pair of the foundations of authority and fairness, anticipating that people’s moral ideology would be moderated by and fluctuate with the market, such that when the economic stress got higher and people were struggling (e.g., from 2007 to 2009), morality would shift to depend on authority; when the economy was on the rise (e.g., after 2009), morality would shift back for fairness. Assuming morality serves the collective but not its individual members, we also report below the extent to which each foundation was endorsed – scored on the raw scale – by individuals and how much the endorsement was directly induced by the depression. The analysis would help separate the interests of the group from those of individual persons, examining whether moral renovation is directed more by the former or by the latter.

Technically, we collected a publicly available, arguably the most immediately and unambiguously felt economic index, namely, the unemployment rate, and individuals’ spontaneous posts on social media (Reddit; see below) for automated text-coding [LIWC-style; 25; see below] in general morality and each of the five moral foundations. The processing allowed us to study the covariation between the society’s economic stress and the population’s level of moralizing each of the foundations. To better cover the recession period, we over-surveyed social-media posts from October, 2007, to the end of 2012. These posts were not topic-limited (i.e., to specific sub-Reddits; see Data Source) and were sorted into months to conform to our pre-set unit of analysis following from that of the unemployment rate (i.e., *monthly* unemployment rate). After text-coding the posts, each month had one score for each foundation and another for general morality, to be matched with a published monthly unemployment rate externally found and linked into the data.

### Data source

We used the open Reddit corpus [26] as the text data source for moral discourse. The social-media platform was where users shared web links to public information and commented on each other’s links. The design likely made the discussions on Reddit originate from and revolve around the public’s reflections of society at the moment, more so than did other popular social media platforms such as Facebook and (the then) Twitter, which were (initially) built for private personal networking. Here, it might be worth noting that, like in the case of all social-media studies, not every Reddit post was recorded in the corpus we used because of users’ privacy settings. Yet, we believe such potential user-opt-out self-selection bias to be relatively ignorable, compared to the same concern on other more-for-personal-use social media, because Reddit was a relatively public forum wherein the comments were meant to go public.

In particular, we included and analyzed all 459,077,063 (> 7-million-per-month) deidentified original and reply posts recorded in the open Reddit corpus during the target 63 months (from October, 2007, to the end of December, 2012). Given the size of the corpus, we did not conduct any data cleaning, believing the decision would preserve the dataset as the most truthful, non-artificially-enhanced image of Reddit we could get. In the meantime, the large sample size should compensate for the statistical power lost in the following noiser analysis. Still, it is important to note that, according to independent surveys, Reddit users were predominantly young males [27], and the largest source country of the users was the United States (see Measures for more details). Lastly, since the data were publicly available and deidentified, the research was not classified as a human-participant study.

## Measures

### Collective economic stress

**Fig 1:**
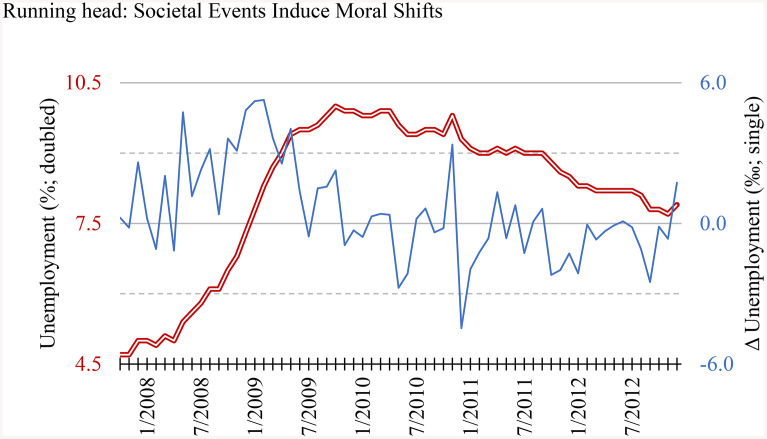
U.S. Unemployment rate and its change rate over the 63 studied months. The red double line indicates the raw monthly unemployment rate against the left axis; the blue single line indicates the rate change against the right axis; note the units are different.

We used the published monthly U.S. unemployment rate (in %; shown in Fig 1) to index economic stress, because unemployment was arguably the most immediately felt economic phenomenon by individuals (cf GDP) and U.S. users comprised the majority (54.4%) of Reddit [28]. The unemployment rates of the second and the third largest source countries of Reddit users – the U.K. (8.2%) and Canada (6.3%) [28] – were also nearly perfectly correlated with that of the U.S. (*r* = .90 and.94 respectively) during our data period, so were captured by the U.S. data too. Indeed, it was indicated that the only three countries that did not enter a recession in 2009 [29] and accounted for > 0.01% traffic on Reddit in 2025 [30] were India, Poland, and S. Korea. These countries together accounted for less than 8.2% traffic in 2025 [30], let alone in 2009, when the website had just been funded in the U.S. for four years. As a result, we were confident that the financial situations of Reddit users could be meaningfully, even if not perfectly, reflected by the U.S. economy in our focused timeframe. Descriptively, as can be seen in the figure, the unemployment rate started rising drastically in mid-2007. The change rate was also generally positive then, indicating monthly increases. In comparison, unemployment slowly but reasonably steadily reduced afterward. The change rate then was mostly negative, thus, a decrease too.

### Morality

We coded moral words in Reddit users’ posts using the Moral Foundations Dictionary [15] for LIWC (Linguistic Inquiry and Word Count; 25) in a LIWC-style procedure, except we programmed our analysis than using LIWC. The dictionary contained eleven validated word categories of the five foundations crossed by two directions of valence – virtue and vice – plus a non-foundation-specific, non-valenced “general morality” category (including words like “moral”). Specifically, we combined virtue and vice within each foundation, because we only focused on whether the foundation was brought into discussion (e.g., in terms of being moral v. amoral) without a prediction for how it was discussed (i.e., moral v. immoral). This also prevented the problem of contextual negation in dictionary-based coding: It is difficult to ascertain whether one *bolsters* or *rejects* a moral *word* when merely the *word* is coded. Together, the processing created six word-categories for the five foundations and general morality. If any word from an MFT dictionary category appeared in a user post, the post was coded 1 as signal-detected; it was coded 0 otherwise. The monthly average signal rates (in %) of the five foundations and general morality were subsequently derived, for testing whether the temporal fluctuations in the five foundation-general morality links tracked with the rise and fall of the economy, the chosen marker of time over the depression (i.e., *when* the economy was going up v. down).

## Results

We first checked the descriptives. As shown in Table 1, at least 14.62% (> 1 in every 6) posts in every covered month were morality-related, indicating morality was a common topic on Reddit. For individual foundations, the mean signal rates also ranged from 4.21% (for purity) to 11.80% (for care), bolstering all five’s substantive existence. Subsequently, because the raw monthly data collected were autocorrelated time series, we applied the differencing technique, creating the monthly *differences* from their corresponding previous months – notated with the symbol Δ – that conformed to the model assumptions of regression. As can be seen in Table 1, all the difference scores of the coded morality variables and the unemployment rate covered both negative and positive scores – going up sometimes and down other times – providing meaningful variability. As such, we fit a planned regression where the monthly Δ general morality was predicted by the five Δ foundations and the Δ unemployment rate – the main effects – and the mean-centered Δ foundation-Δ unemployment interactions – the key moderated effects [After inspecting the ACF and the PACF plots, we also fit an ARIMA(1,1,1) model to the data, namely, adding an AR(1) and an MA(1) to the model decribed in the main text. The results indicated that both the AR and the MA effects were non-significant, and the rest of the effects showed the same pattern, with only the Δ fairness-x-Δ unemployment interaction dropped to marginal significance at *p* = .061.]. Here, we used these predictors altogether, so their uniqueness was estimated.

**Table 1:** Descriptives of key variables.

Variable	Min	M	Max	SD
Unemployment	4.706	8.171	9.983	1.617
General morality	14.621	16.194	17.477	0.586
Care	9.826	11.800	14.896	1.344
Fairness	3.794	4.578	5.844	0.471
Loyalty	5.015	6.222	8.306	0.922
Authority	6.991	8.664	11.616	1.237
Purity	3.707	4.214	4.871	0.269
Δ Unemployment	−0.447	0.051	0.527	0.210
Δ General morality	−2.430	−0.028	2.113	0.553
Δ Care	−1.913	−0.054	1.942	0.666
Δ Fairness	−0.599	−0.028	0.680	0.223
Δ Loyalty	−0.932	−0.049	1.076	0.292
Δ Authority	−1.741	−0.072	1.409	0.406
Δ Purity	−0.581	−0.008	0.874	0.222

All variables are in %.

**Fig 2:**
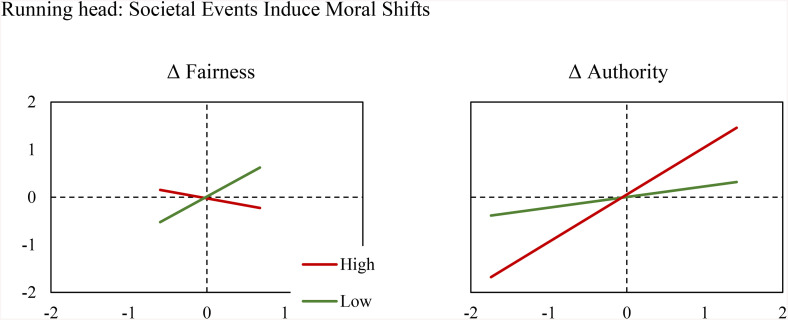
Δ General Morality Predicted (Y) by Δ Fairness and Δ Authority (X) given High and Low Δ Unemployment. High (0.261%, red) and low (−0.159%, green) Δ unemployment rates are defined as M ± SD; the lines are from the lowest to the highest Δ fairness and Δ authority in the data; Δ fairness varied less, so did its effect on Δ general morality, so its figure (left) appears smaller than that of Δ authority (right).

Supporting the hypotheses, results in Table 2 and Fig 2 show Δ unemployment was significantly positively associated with the predictability of Δ authority for Δ general morality and significantly negatively associated with that of Δ fairness. That is, the higher the jump in unemployment in a month, the larger the increase in authority’s contribution to Δ general morality and the smaller that of fairness in the same month. On the contrary, Δ unemployment was not associated with the predictability of Δ care, Δ loyalty, or Δ purity. The degree of change in unemployment in a month did not correspond to the difference in these three domains of morality.

**Table 2:** Results of the main analysis predicting δ general morality.

Predictor	B	SE	*t*	*p*	CI 95% bounds	
					Lower	Upper
Intercept	0.06	0.04	1.36	.179	−0.03	0.15
Δ Unemployment	−0.01	0.22	−0.07	.946	−0.45	0.42
Δ Care	−0.11	0.10	−1.10	.279	−0.32	0.10
x Δ Unemployment	0.46	0.37	1.23	.225	−0.29	1.20
Δ Fairness	0.30	0.29	1.05	.299	−0.28	0.88
x Δ Unemployment*	−2.84	1.18	−2.41	.020	−5.22	−0.47
Δ Loyalty	0.38	0.23	1.67	.101	−0.08	0.84
x Δ Unemployment	−0.97	0.94	−1.04	.305	−2.86	0.92
Δ Authority*	0.61	0.20	3.09	.003	0.22	1.01
x Δ Unemployment*	1.84	0.71	2.61	.012	0.42	3.26
Δ Purity*	1.04	0.36	2.89	.006	0.32	1.77
x Δ Unemployment	−2.71	1.53	−1.77	.083	−5.79	0.37

*Note*: * indicates *p* <.05; focused tests are underscored.

Looking closely, the simple slopes of Δ authority and Δ fairness in the months wherein the most individuals were fired (Feb. 2009) and hired (Oct. 2010) suggest the change in the use of general morality words was significantly and positively attributable to that of authority (B = 1.48, *t* = 4.52, *df* = 50, *p* < .001) but not fairness in the most stressful month (B = −1.03, *t* = −2.00, *df* = 50, *p* = .051). The effec*t* of fairness almost became significant in the negative direction. In contrast, only Δ fairness (B = 0.84, *t* = 4.44, *df* = 50, *p* < .001) bu*t* not Δ authority (B = −0.30, *t* = −0.67, *df* = 50, *p* = .508) appeared significantly and posi*t*ively associated with Δ general morality in the least stressful month. In short, authority and fairness were entirely silenced in the most and the least employable months, respectively, leaving the other on stage to monologize on what morality was.

### Supplementary analyses

As mentioned, we note that there were two significant positive main effects of Δ foundations on Δ general morality. One was of Δ authority and thus moderated by time (i.e., Δ unemployment). The other was of Δ purity and not moderated. This finding of Δ purity might indirectly supplement the overarching hypothesis by indicating that a foundation would persist and not change in its influence on defining what is moral v. amoral when the society shifts in a way that has little to do with the foundation.

We also explored temporal changes in the foundations and show the results in Table 3. Because time was marked by Δ unemployment rate, we regressed each of the five Δ foundations, one at a time, on Δ unemployment rate, the time variable here, controlling for the other four Δ foundations. The results showed no direct predictability of Δ unemployment rate of any Δ foundation. The finding bolstered the idea that endorsing a foundation is not the same as linking it to the broader conception of morality.

**Table 3:** Results of the supplementary analyses predicting each δ moral foundation.

Predictor	B	SE	*t*	*p*	CI 95% bounds (%)	
					Lower	Upper
Intercept → Δ Care	0.00	0.07	0.01	.991	−0.13	0.14
Δ Unemployment	0.24	0.31	0.75	.457	−0.39	0.87
Intercept → Δ Fairness	−0.01	0.02	−0.49	.629	−0.06	0.03
Δ Unemployment	0.06	0.11	0.59	.559	−0.15	0.27
Intercept → Δ Loyalty	−0.01	0.03	−0.29	.775	−0.06	0.05
Δ Unemployment	−0.16	0.13	−1.27	.210	−0.42	0.09
Intercept → Δ Authority	−0.03	0.04	−0.82	.415	−0.10	0.04
Δ Unemployment	0.00	0.17	−0.01	.992	−0.34	0.34
Intercept → Δ Purity	0.01	0.02	0.63	.532	−0.03	0.06
Δ Unemployment	0.05	0.10	0.53	.601	−0.15	0.25

All four non-dependent-variable foundations are controlled for; their statistics are in the supplementary materials (Supplementary S1–S5 Tables); no effect here is significant.

## Discussion

The current paper reasons and provides partial correlational evidence for the hypothesis that large-scale societal events induce revisions in individual moral ideology in the direction that the incoming moral conception would supposedly help the people survive their shared new socio-eco-challenges. Using a gigantic naturalistic social-media record (N = 459,077,063) over the last global recession (63 months from October, 2007, to the end of 2012), we discover the economic havoc was associated with increased *moral* weight of authority and decreased moral weight of fairness. When the economy was reviving, the public’s morality shifted back from authority to again lean on fairness. This pair of antagonistic effects serves as a demonstration of our overall theorizing, in line with our theory that morality is a group-level evolutionary product that functions to hold the collective together.

Here, it is critical to emphasize we are not merely talking about people’s regular concerns with authority and fairness per se, but the degrees to which these dispositions are moralized as *ethical* concerns. One may well obey authorities due to personal fear, perceiving no legitimacy in them. Both motivations are possible, but only the latter is moral. In the chosen example, when the economy was falling, a heightened belief in authority being a *moral* issue would potentially license the society to officially enforce the attitude, consequently justifying the then steep class hierarchy and preventing anyone from breaking it apart [31,32]. Likewise, when the economy was rising and there was a surplus in production, an increased moral value of fairness would not only encourage but indeed mandate the resources be distributed across the public. Both of these would benefit the collective in their times: The society got to sustain its existence, even though some individuals might be sacrificed for the greater good [31].

### Implications

For theory, the present work speaks to the potential need to parse out personal and public preferences as separate constructs [31], with moral ideology (also) belonging to the group but not (only) to individuals. This distinction of the two layers of preferences is analogous to Billing’s classic observation that the behavior of thinking and arguing for the thought is not a mere private individual action; it rests on and manifests a public ideology shared by many, even if the thinker is not aware of such a macro-to-micro, ours-to-mine connection. [33]; see also Sammut’s work on how seemingly personal common sense represents socially negotiated inter-objectivity, [34] Similarly, the present paper defines a personal preference as what individual members of the group wish for themselves. A heterosexual person may prefer a different-sex partner for themselves, deeming the preference personal, hence having no trouble supporting others’ same-sex marriages. A public preference of the people, on the other hand, is what they expect the entire group – every member constituting the unity and its culture – to follow. A heterosexual person who holds their sexual orientation moral, thus public beyond individual would accordingly not only pursue a different-sex partner for themselves, but also demand the attitude be enforced for all, say, through the law.

Echoing this contrast, we document that people in the recession did not associate fairness more with their general conviction of what the abstract idea of morality entailed, when a fairer economy could have been especially conducive to personal self-interest, enjoyable, and even necessary for some. Instead, it was the pursuit of a more hierarchically structured and stable/stabilized collective that gained its *moral* momentum. The results, therefore, lend support for morality being a public preference, as it was the people as a singular totality that would supposedly benefit from the attitude. People, as a plural, might actually suffer as the oblations. Empirically, individual persons’ agreements with the five moral foundations per se did not track with the five’s unfolding trajectories of moralization either. Overall, our work suggests personal moral dispositions, though expressed by individuals, mirror the collective’s common social and cultural surroundings, too. As such, the dispositions may move with the group’s changing fate, gradually yet more or less uniformly.

Methodologically, the phenomenon also points to a need to reconsider how to measure moral dispositions properly. A potentially fitting analogy is that assessing morality multiple times in a shifting environment, as we did in the present study, resembles using a questionnaire cross-sectionally but in multiple cultures. With the latter ubiquitous research design, the researchers usually know to test the measurement invariance of the scale – that it taps into the same construct cross-culturally [e.g., 35,36]. Only with the invariance set up can one advance to score each culture using the measure. Otherwise, it becomes equivocal to compare the orange found in one place to the apple in another. Yet, when it comes to cultural changes, which similarly concern a varying cultural background, it somehow becomes acceptable that the invariance does not need to be confirmed. The questionnaire would be assumed to be cross-temporally invariant, to which we document counter-evidence here in the moral domain. Future investigations into moral personalities and maybe all personalities alike would consequently want to stay cautious and serious about this methodological pitfall, especially with one-shot measurements where there is still one, thus easily overlooked context hidden behind.

Lastly, an alternative interpretation of the current research concerns the specificity of the five moral foundations: The results might reveal that people simply turn collectivist and unite together during crises [37]. This is because, in the MFT literature, some scholars classify care and fairness as individualizing foundations, for they require people to judge one another as individual persons and treat them as such [e.g., 8]. In comparison, loyalty, authority, and purity are called binding foundations, which feed on and for group-based cognition and cohesion. Ostensibly, the two classes of foundations correspond to individualism and collectivism, respectively, leading to the suggestion that our study shows the society shifts away from individualism to collectivism in economic hardship. Yet, we would point out that each of both classes of the foundations has more than one element, but only one from each class – fairness from the individualizing and authority from the binding set – appeared to be predictive over the surveyed depression. This provides preliminary support that the two focal foundations are effectively discernable, therefore conceptually unique against their same-class elements.

### Limitations and future directions

With its strengths, there are limitations in the current work. First, although the study was based on 459,077,063 public comments non-intrusively collected over 63 months, the comments were retrieved from a single platform, while all social media have their own cultures [38]. Among them, Reddit users are known for being predominantly young males in English-speaking countries [27]. Although this contributed to our confidence in the internal validity of using the *U.S.* unemployment rate as the economic index, it might have decreased the external validity of extending our conclusion to other places and demographics. On a related note, although we attribute the moral renovation found on Reddit to the larger fluctuating economic environment, it could be the case that the renovation was caused by a change in the Reddit user demographics. We, however, do not think this was too much of a concern, because demographic changes on social media within a few years (e.g., five, as in our case) should more commonly be unidirectional. In the case of Reddit, it has been reported that the platform gradually became more global and less male-dominant over the past decade or so. The change was slow, much slower than potentially having moved in one direction and then back within the five years of our data, to be a corresponding factor that may explain the morality shifting back *and forth* in our study. Likewise, research shows that people speak and think differently online and offline. [39] Future investigations would hence want to utilize and compare distinct data sources and types to examine the generalizability of our research.

Secondly, our findings only represent a special case of the proposed phenomenon of moral renovation, namely, that between large-scale unemployment and the foundations of authority and fairness. Future work should therefore examine alternative measures of economic stress, even non-economic societal events, thus their associations with different collective needs and moral foundations. For instance, we applied a similar text analysis in an unpublished study on President Bush’s State-of-the-Union addresses to Congress in the years before and after the 911 attacks. Unsurprisingly, the results showed that the foundation of loyalty – that functions to separate us from them – was moralized only, but very much so post-attack in 2002. In 2001, “the right thing to do” in the speech was, instead, all internal affairs, including tax cuts, Medicare, Social Security, education, and the environment. National defense was merely lightly and abstractly mentioned on the side as something that “may” need to be “review[ed]” for “the next ten years.” There are also newly discovered foundations to be considered [36], though we could not incorporate them because they were not yet in the dictionary we applied.

For a deeper understanding of the inner work of moral renovation, scholars may try to look further into the social mechanisms that helped realize the moral renovation we report and other instances of such renovation in future investigations. In the present research, we relied on online text analysis, therefore jumping directly from the level of societal economic happenings (as marked by objective time) to that of interpersonal discourses. Nevertheless, there should have been everyday material processes that practically linked and step-by-step brought the macro beginning to the micro end, as argued by theorists such as Billing [33] and Sammut [34]. For example, one may examine how the political dynamics, ranging from public speeches from government officials, debates on the Congress floor, to policies administered to the people, might have served to reveal to the public the crisis facing the collective, as well as how the challenge could be better responded to (e.g., then-President Obama emphasized the importance to stabilize the economy in his address to the Congress). Likewise, toward the end of our data period, where the market was recovering, grassroots movements such as Occupy Wall Street mgith have assisted not only in informing the members of the society about the economy, but also in stimulating a rething of what a moral group should look like (e.g., to treat “we the 99%” equally as the top 1%).

Similarly, researchers may consider experimentally inducing the perception of cultural shifts in moral norms in the laboratory, for a stronger causal inference than that in our correlational study. We are, however, uncertain how much time is required for the proposed moral renovation to ferment and take effect for personalities that are as consistent as moral ideology, thus whether the process would fit into the temporal scope of experiments. In the present demonstration, we operated on the scale of months to years, and it seems a full-fledged experiment would need to radically shrink the scope to hours or at least days. We believe the work would be challenging but, at the same time, not entirely impossible depending on the intervention techniques [40], and surely an exciting opportunity to shed light on the nature of moral cultural development.

Finally, we measured Reddit users’ naturalistic discourse focused on various aspects of morality, but the discourse might have been built from different, pro- and anti-perspectives. People often say one thing and do another, too [41]. Here, our analytical decision stemmed from the assumption that morality at the foundational level can only be amoral but not immoral [15,16]. However, it is debatable whether the foundations the field has offered are already foundational. If they are not, we might have lost the nuanced moral tones in which people wrote about morality. Beyond morality, it is also known that a promotion (e.g., of virtues) and a prevention (e.g., of vices) focus on behavior for the same goal (e.g., for the same moral functions) may lead to different results [42]. It is consequently recommended that individuals’ concrete actions on – for and against – moral principles be assessed whenever feasible. For instance, though not creating group loyalty directly by oneself, one’s voting for a candidate campaigning for group distinction and separation could be an effective behavioral indicator of one’s leaning towards moral loyalty. Such a research design shall not only speak louder than theoretical words, but also open the applied opportunities for understanding real-world matters (e.g., polarization: Could it be that polarization exacerbates because people focus not only on different moral foundations but also on different ways to focus on the foundations?) and devising collective moral interventions for them.

## Supporting information

S1 Table Full results of the supplementary analysis predicting δ care. * indicates p < .05.
(DOCX)

S2 Table Full results of the supplementary analysis predicting δ fairness. * indicates p < .05.
(DOCX)

S3 Table Full results of the supplementary analysis predicting δ loyalty. * indicates p < .05.
(DOCX)

S4 Table Full results of the supplementary analysis predicting δ authority. * indicates p < .05.
(DOCX)

S5 Table Full results of the supplementary analysis predicting δ purity. * indicates *p* < .05.
(DOCX)
