# Supplementary material for: Under renovation: Large-scale societal events induce shifts between moral ideologies
Source: PLoS One. 2025 Dec 10;20(12):e0336520. doi: 10.1371/journal.pone.0336520 (PMC12694803; doi:10.1371/journal.pone.0336520)
Supplement: S1 Table — * indicates p < .05. (DOCX) [file pone.0336520.s001.docx]

| S1 Table. Full Results of the Supplementary Analysis Predicting Δ Care | | | | | | |
| --- | --- | --- | --- | --- | --- | --- |
| Predictor | B | SE | *t* | *p* | CI 95 bounds | |
|  |  |  |  |  | Lower | Upper |
| Intercept | 0.00 | 0.07 | 0.01 | .991 | −0.13 | 0.14 |
| Δ Unemployment | 0.24 | 0.31 | 0.75 | .457 | −0.39 | 0.87 |
| Δ Fairness | −0.66 | 0.39 | −1.69 | .096 | −1.44 | 0.12 |
| Δ Loyalty * | 1.04 | 0.29 | 3.56 | .001 | 0.45 | 1.62 |
| Δ Authority | 0.38 | 0.24 | 1.58 | .120 | −0.10 | 0.86 |
| Δ Purity | 0.77 | 0.41 | 1.89 | .064 | −0.05 | 1.58 |
| *Note*: * indicates *p* < .05. | | | | | | |
