# Supplementary material for: Under renovation: Large-scale societal events induce shifts between moral ideologies
Source: PLoS One. 2025 Dec 10;20(12):e0336520. doi: 10.1371/journal.pone.0336520 (PMC12694803; doi:10.1371/journal.pone.0336520)
Supplement: S2 Table — * indicates p < .05. (DOCX) [file pone.0336520.s002.docx]

| S2 Table. Full Results of the Supplementary Analysis Predicting Δ Fairness | | | | | | |
| --- | --- | --- | --- | --- | --- | --- |
| Predictor | B | SE | *t* | *p* | CI 95 bounds | |
|  |  |  |  |  | Lower | Upper |
| Intercept | −0.01 | 0.02 | −0.49 | .629 | −0.06 | 0.03 |
| Δ Unemployment | 0.06 | 0.11 | 0.59 | .559 | −0.15 | 0.27 |
| Δ Care | −0.07 | 0.04 | −1.69 | .096 | −0.16 | 0.01 |
| Δ Loyalty | 0.17 | 0.11 | 1.59 | .117 | −0.04 | 0.38 |
| Δ Authority * | 0.17 | 0.08 | 2.14 | .036 | 0.01 | 0.33 |
| Δ Purity * | 0.42 | 0.13 | 3.25 | .002 | 0.16 | 0.67 |
| *Note*: * indicates *p* < .05. | | | | | | |
