# Supplementary material for: Under renovation: Large-scale societal events induce shifts between moral ideologies
Source: PLoS One. 2025 Dec 10;20(12):e0336520. doi: 10.1371/journal.pone.0336520 (PMC12694803; doi:10.1371/journal.pone.0336520)
Supplement: S3 Table — * indicates p < .05. (DOCX) [file pone.0336520.s003.docx]

| S3 Table. Full Results of the Supplementary Analysis Predicting Δ Loyalty | | | | | | |
| --- | --- | --- | --- | --- | --- | --- |
| Predictor | B | SE | *t* | *p* | CI 95 bounds | |
|  |  |  |  |  | Lower | Upper |
| Intercept | −0.01 | 0.03 | −0.29 | .775 | −0.06 | 0.05 |
| Δ Unemployment | −0.16 | 0.13 | −1.27 | .210 | −0.42 | 0.09 |
| Δ Care * | 0.18 | 0.05 | 3.56 | .001 | 0.08 | 0.28 |
| Δ Fairness | 0.26 | 0.16 | 1.59 | .117 | −0.07 | 0.58 |
| Δ Authority * | 0.23 | 0.10 | 2.39 | .020 | 0.04 | 0.43 |
| Δ Purity | −0.06 | 0.17 | −0.34 | .735 | −0.40 | 0.29 |
| *Note*: * indicates *p* < .05. | | | | | | |
