# Supplementary material for: Under renovation: Large-scale societal events induce shifts between moral ideologies
Source: PLoS One. 2025 Dec 10;20(12):e0336520. doi: 10.1371/journal.pone.0336520 (PMC12694803; doi:10.1371/journal.pone.0336520)
Supplement: S4 Table — * indicates p < .05. (DOCX) [file pone.0336520.s004.docx]

| S4 Table. Full Results of the Supplementary Analysis Predicting Δ Authority | | | | | | |
| --- | --- | --- | --- | --- | --- | --- |
| Predictor | B | SE | *t* | *p* | CI 95 bounds | |
|  |  |  |  |  | Lower | Upper |
| Intercept | −0.03 | 0.04 | −0.82 | .415 | −0.10 | 0.04 |
| Δ Unemployment | 0.00 | 0.17 | −0.01 | .992 | −0.34 | 0.34 |
| Δ Care | 0.11 | 0.07 | 1.58 | .120 | −0.03 | 0.25 |
| Δ Fairness * | 0.45 | 0.21 | 2.14 | .036 | 0.03 | 0.86 |
| Δ Loyalty * | 0.40 | 0.17 | 2.39 | .020 | 0.07 | 0.73 |
| Δ Purity * | 0.46 | 0.22 | 2.11 | .039 | 0.02 | 0.90 |
| *Note*: * indicates *p* < .05. | | | | | | |
