# Supplementary material for: Under renovation: Large-scale societal events induce shifts between moral ideologies
Source: PLoS One. 2025 Dec 10;20(12):e0336520. doi: 10.1371/journal.pone.0336520 (PMC12694803; doi:10.1371/journal.pone.0336520)
Supplement: S5 Table — * indicates p < .05. (DOCX) [file pone.0336520.s005.docx]

| S5 Table. Full Results of the Supplementary Analysis Predicting Δ Purity | | | | | | |
| --- | --- | --- | --- | --- | --- | --- |
| Predictor | B | SE | *t* | *p* | CI 95 bounds | |
|  |  |  |  |  | Lower | Upper |
| Intercept | 0.01 | 0.02 | 0.63 | .532 | −0.03 | 0.06 |
| Δ Unemployment | 0.05 | 0.10 | 0.53 | .601 | −0.15 | 0.25 |
| Δ Care | 0.08 | 0.04 | 1.89 | .064 | 0.00 | 0.16 |
| Δ Fairness * | 0.38 | 0.12 | 3.25 | .002 | 0.15 | 0.62 |
| Δ Loyalty | −0.04 | 0.10 | −0.34 | .735 | −0.24 | 0.17 |
| Δ Authority * | 0.16 | 0.08 | 2.11 | .039 | 0.01 | 0.31 |
| *Note*: * indicates *p* < .05. | | | | | | |
